# SARS-CoV-2 B.1.1.7 Variant Infection in Malayan Tigers, Virginia, USA

**DOI:** 10.3201/eid2712.211234

**Published:** 2021-12

**Authors:** Patrick K. Mitchell, Mathias Martins, Tara Reilly, Leonardo C. Caserta, Renee R. Anderson, Brittany D. Cronk, Julia Murphy, Erin L. Goodrich, Diego G. Diel

**Affiliations:** Cornell University, Ithaca, New York, USA (P.K. Mitchell, M. Martins, L.C. Caserta, R.R. Anderson, B.D. Cronk, E.L. Goodrich, D.G. Diel);; Virginia Zoo, Norfolk, Virginia, USA (T. Reilly);; Virginia Department of Health, Richmond, Virginia (J. Murphy)

**Keywords:** SARS-CoV-2, respiratory infections, severe acute respiratory syndrome coronavirus 2, SARS, COVID-19, coronavirus disease, zoonoses, viruses, coronavirus, tigers, zoonoses, One Health, Virginia, United States

## Abstract

We report infection of 3 Malayan tigers with severe acute respiratory syndrome coronavirus 2 (SARS-CoV-2) B.1.1.7 (Alpha) variant at a zoologic park in Virginia, USA. All tigers exhibited respiratory signs consistent with SARS-CoV-2 infection. These findings show that tigers are susceptible to infection with the SARS-CoV-2 B.1.1.7 variant.

On April 4, 2021, a 5-year-old male Malayan tiger (*Panthera tigris jacksoni*) at the Virginia Zoo (Norfolk, VA, USA) began exhibiting lethargy, labored breathing, coughing, intermittent upper respiratory sounds, hyporexia, and mucoid nasal discharge. On April 7, another 5-year-old male Malayan tiger began experiencing labored breathing, cough, clear nasal discharge, and hyporexia. On April 10, a third Malayan tiger, a 10-year-old male, had cough and later clear nasal discharge. The tigers’ clinical signs resolved by April 15, eleven days after the outbreak began.

Zoo staff collected nasal swab and fecal samples from the 5-year-old tigers on April 9 and the 10-year-old tiger on April 13 and submitted these to Cornell University’s Animal Health Diagnostic Center (AHDC; Ithaca, NY, USA). AHDC tested samples for *Bordetella* sp., *Chlamydia felis*, *Mycoplasma cynos*, *M. felis*, *Streptococcus equi* subspecies *zooepidemicus*, influenza virus, pneumovirus, feline calicivirus, and feline herpesvirus; all results were negative. All samples tested positive for severe acute respiratory syndrome coronavirus 2 (SARS-CoV-2) by EZ-SARS-CoV-2 Real-Time RT-PCR Test (Tetracore, Inc., https://tetracore.com). We isolated SARS-CoV-2 from respiratory and fecal specimens from the first tiger. Testing at the US Department of Agriculture National Veterinary Services Laboratories (Ames, IA, USA) confirmed SARS-CoV-2 infection. We screened the tiger samples using TaqPath COVID-19 RT-PCR Kit (Thermo Fisher Scientific, https://www.thermofisher.com), which revealed a spike gene dropout in samples from all 3 tigers; only the nucleoprotein and open reading frame 1ab gene targets were detected, suggesting B.1.1.7 variant infection.

We performed whole-genome sequencing on all samples by using MinION (Oxford Nanopore Technologies, https://nanoporetech.com), as previously described (*1*). We assembled reads using the ARTIC ncov-2019 protocol (ARTIC Network, https://artic.network) and Medaka (Oxford Nanopore Technologies) for variant calling. We obtained near-complete (29,702–29,710-bp) assemblies from all nasal swab specimens (GenBank accession nos. MZ305031–3) but no assemblies from fecal samples. We identified respiratory specimen genomes as lineage B.1.1.7 (Alpha variant) by using Pangolin version 2.4.2 (https://github.com/cov-lineages/pangolin). We used Nextstrain (https://nextstrain.org) for phylogenetic analysis of tiger-derived sequences and other B.1.1.7 sequences downloaded from GISAID (https://www.gisaid.org) on April 15, 2021 (*2*,*3*). Tiger-derived sequences all were identical, except 1 manually corrected homopolymer repeat error, and fell into a clade defined by a C4900T mutation containing other samples collected primarily in the United States. Tiger-derived sequences differed from others in the clade by 1 single-nucleotide polymorphism in the spike gene (K558N) (Figure, panel A). Using the vdb tool (*4*), we found 46 additional B.1.1.7 sequences that had the K558N mutation in GISAID on July 22, 2021; all were collected from Virginia during March 27–July 7, 2021. However, phylogenetic analysis of these sequences and the tiger-derived sequences showed divergence of 11 single-nucleotide polymorphism, minus the divergence producing the K558N mutation (Figure, panel B), indicating the sequences are not related epidemiologically.

**Figure:**
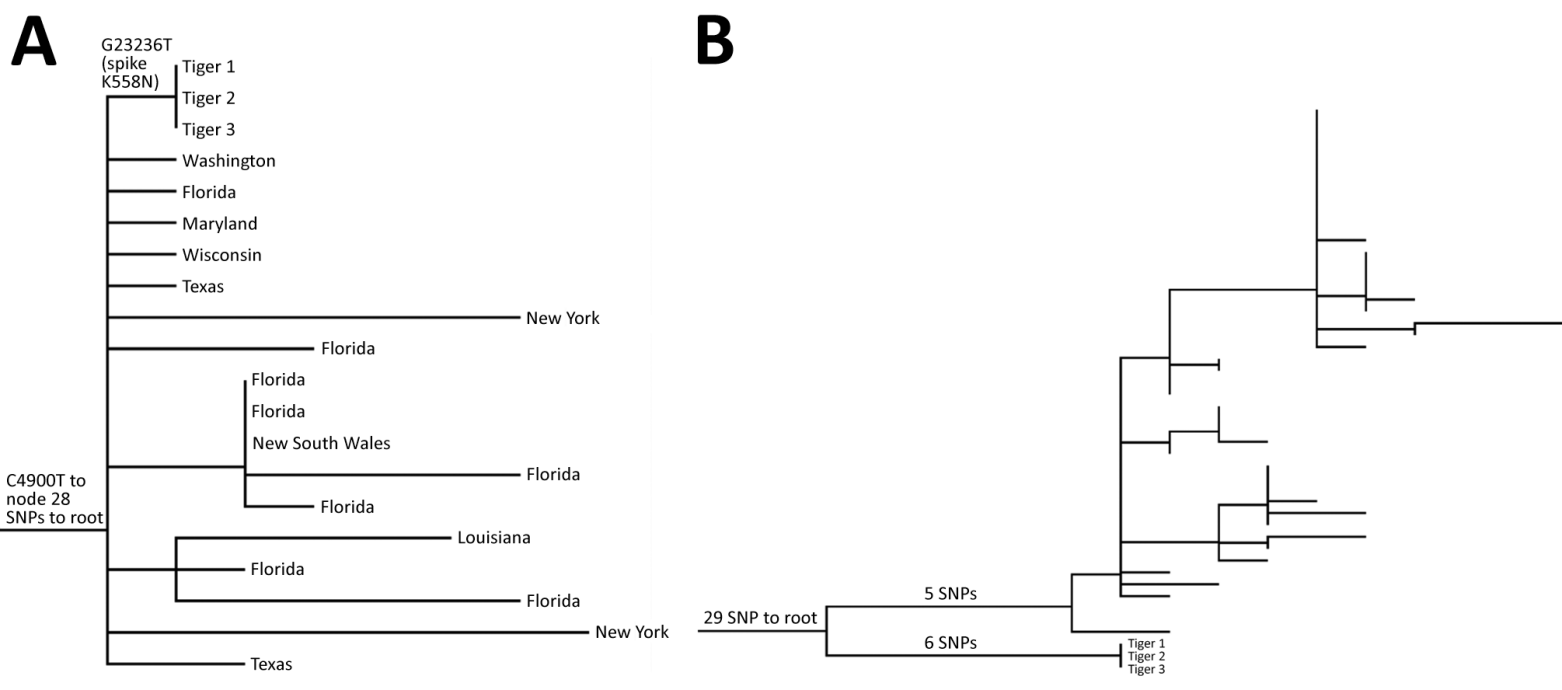
Maximum-likelihood phylogenetic trees of severe acute respiratory syndrome coronavirus 2 from 3 Malayan tigers, Virginia, USA. Tiger samples are numbered in order of symptom onset. A) Subset of phylogenetic tree showing parent (G23236T) and grandparent (C4900T) nodes of the tiger sequences, with tips labeled as states of origin in the United States or Australia. B) Phylogenetic tree showing that other B.1.1.7 viruses detected in Virginia that contain the K558N mutation are not epidemiologically related to the sequences detected in tigers 1, 2, and 3. SNP, single-nucleotide polymorphism.

The source of the tigers’ infection is unknown. The zoo has been open to the public, but transmission from a visitor is unlikely because tiger exhibit areas are separated from visitors by either a glass enclosure or >9 m distance. The most plausible explanation is that >1 tiger acquired the virus from a keeper because they had close contact. However, no employees tested positive for SARS-CoV-2 nor had symptoms during the 4 weeks before the tigers’ symptom onset. Nine keepers were responsible for the animals’ daily care; 2 other persons prepared animal diets daily. Employees were required to wear facemasks always, indoors and outdoors; everyone wore standard 2-ply surgical masks or homemade cloth facemasks. Staff also were required to wear gloves when handling and preparing food and when servicing animal areas. Furthermore, staff were required to step into an accelerated hydrogen peroxide disinfectant footbath when entering the tiger building and diet kitchen. The 3 tigers might have been infected by an employee, or 1 tiger was infected, then transmission occurred to the others. Two tigers lived in the same enclosure and had no direct contact with the third, but all 3 rotated through common enclosure spaces.

After identification of the tiger infections, 4 additional zoo animals were tested: 1 lion (*Panthera leo*) with lethargy and hyporexia ≈1 week after SARS-CoV-2 diagnosis in the tigers; another asymptomatic lion because of age and proximity to the first lion; and 2 degus (*Octodon degus*) that died in late March and had interstitial pneumonia on necropsy. AHDC tested nasal swab samples from the lions and frozen spleen and cecum samples from the degus by reverse transcription PCR; all results were negative for SARS-CoV-2.

Our findings underscore felid susceptibility to SARS-CoV-2, which also has been detected in captive snow leopards (*Panthera uncia*) and pumas (*Puma concolor*) (*5*). Other nonhuman species, including gorillas (*Gorilla gorilla*), minks (*Neovison vison*), and ferrets (*Mustela putorius furo*), have acquired SARS-CoV-2; additional species have been shown to be susceptible experimentally (*5**–**7*). Domestic cats and dogs in the United Kingdom and United States reportedly had B.1.1.7 infections, suggesting that mutations characterizing this lineage are not constrained to a host range (*8*; L. Ferasin et al., unpub. data, https://doi.org/10.1101/2021.03.18.435945). Monitoring animals for SARS-CoV-2 infection is critical to determining potential host range, particularly as new virus variants emerge and spread.
